# A Scoping Review of Clinical Guidelines for the Management of Cardiovascular Diseases (CVD) in Pregnancy in Low- and Middle-Income Countries (LMIC)

**DOI:** 10.5334/gh.1453

**Published:** 2025-08-21

**Authors:** Uma Vasudevan, Preety Rajbangshi, Jane Hirst

**Affiliations:** 1 The George Institute for Global Health, IN; 2 The George Institute for Global Health, UK

**Keywords:** Cardiovascular diseases, low and middle-income countries, hypertensive disorders of pregnancy, preeclampsia, women’s health

## Abstract

Cardiovascular diseases (CVD), including pre-existing cardiac conditions and hypertensive disorders of pregnancy, are among the leading causes of maternal mortality globally and account for a substantial proportion of preventable deaths in low- and middle-income countries (LMICs). In these settings, women are disproportionately affected by conditions such as rheumatic heart disease, peripartum cardiomyopathy, and severe anemia-related heart failure, yet clinical guidance tailored to LMICs contexts remains limited. This paper presents the findings of a scoping review on CVD in pregnancy guidelines in LMICs. The review seeks to identify and map clinical guidelines from LMICs and international organizations with reach in LMICs that addresses the prevention, screening, and management of cardiovascular diseases (CVD) in pregnancy and examine their scope, content, and specific recommendations for pregnant women. The review followed the JBI methodology. Guidelines on CVD care in pregnancy published between 2011 and 2023 by international or national professional organizations and Ministries of Health from LMICs were searched using databases such as PubMed, Scopus, GIN International library, and individual websites. Data were extracted using a custom-designed MS Excel form, capturing details such as guideline title, year, type, publisher, country, target audience and population, clinical focus, timing, and summary of recommendations. Out of the 90 shortlisted guidelines, 17 were included and 73 were excluded. Of the included guidelines, 3 are on CVDS and 14 are on hypertensive disorders of pregnancy (HDP). They varied in scope, with most focusing on preeclampsia or HDP, but only a few provided comprehensive recommendations across the continuum of cardiovascular care in pregnancy, highlighting major gaps in prevention, screening, and long-term follow-up. Existing guidance remains fragmented with limited coverage of high-burden conditions of LMICs such as rheumatic heart disease. Strengthening clinical practice will require not only adapting global recommendations to local realities but also investing in LMIC-led research and inclusive guideline development that reflects regional priorities and health system capacities.

## Introduction

The World Health Organization (WHO) estimates that over 95% of maternal deaths occur in low- and middle-income countries (LMIC), the majority of which are preventable (1). Cardiovascular diseases (CVD) account for one in three pregnancy-related maternal deaths, making them the single largest cause of indirect maternal mortality (2,3). CVD in pregnancy includes pre-existing cardiac conditions (congenital or acquired), other diseases of the circulatory system such as chronic hypertension, cerebrovascular disease, and coronary artery disease, as well as cardiovascular conditions that arise during pregnancy. These include hypertensive disorders of pregnancy (HDP)—a spectrum comprising ‘pre-existing hypertension, gestational hypertension, preeclampsia, chronic hypertension with superimposed preeclampsia, and antenatal unclassifiable hypertension’ (4)—and distinct cardiac conditions such as valvular lesions or peripartum cardiomyopathy (5). Pre-existing cardiac conditions are a significant cause of maternal morbidity and mortality, affecting between 1% and 4% of all pregnancies (6). Among these women, 16% will develop cardiac complications such as arrhythmia and heart failure during the course of pregnancy (7), and 2%–8% will acquire hypertensive disorders of pregnancy (HDP). Notably, 14% of maternal deaths are due to these conditions (8). Moreover, CVD in pregnancy disproportionately affects women from or living in low- and middle-income countries, as well as black and people of color living in all settings (9,10,11,12). Some conditions, such as rheumatic heart disease, are associated with childhood poverty, whilst other conditions are related to diet and nutrition. For example, in India, anemia is the most common cause of heart failure in pregnancy (13). It is estimated that one in four maternal deaths in Latin America and one in ten in Asia and Africa are due to complications from CVD (14).

To prevent these deaths, detection of women at risk before, during, and after pregnancy is essential, along with multidisciplinary care during pregnancy and after delivery from obstetric and cardiology care providers (15). While numerous guidelines have been developed by global and national professional bodies to address various aspects of CVD and HDP, there has been no prior comprehensive mapping of the availability, scope, and relevance of these guidelines for LMIC contexts.

This review looks at the clinical guidelines on CVD in pregnancy for LMIC settings. It answers the following questions:

- What clinical guidelines or policies from LMICs have been developed to address screening, prevention, and management of women with CVD before, during, and after pregnancy?
- How do these guidelines differ in their recommendations for (i) the prevention of preeclampsia, (ii) the management of hypertensive disorders of pregnancy, and (iii) the management of women with cardiac diseases during pregnancy?

This paper identified and mapped clinical guidelines highlighting the gaps in coverage and significant variations in preventative and therapeutic management recommendations within the guidelines. This paper further provides recommendations to improve the provision of evidence-based care for pregnant women with CVD in LMICs.

## Methodology

### Review methodology

**Figure 1:**
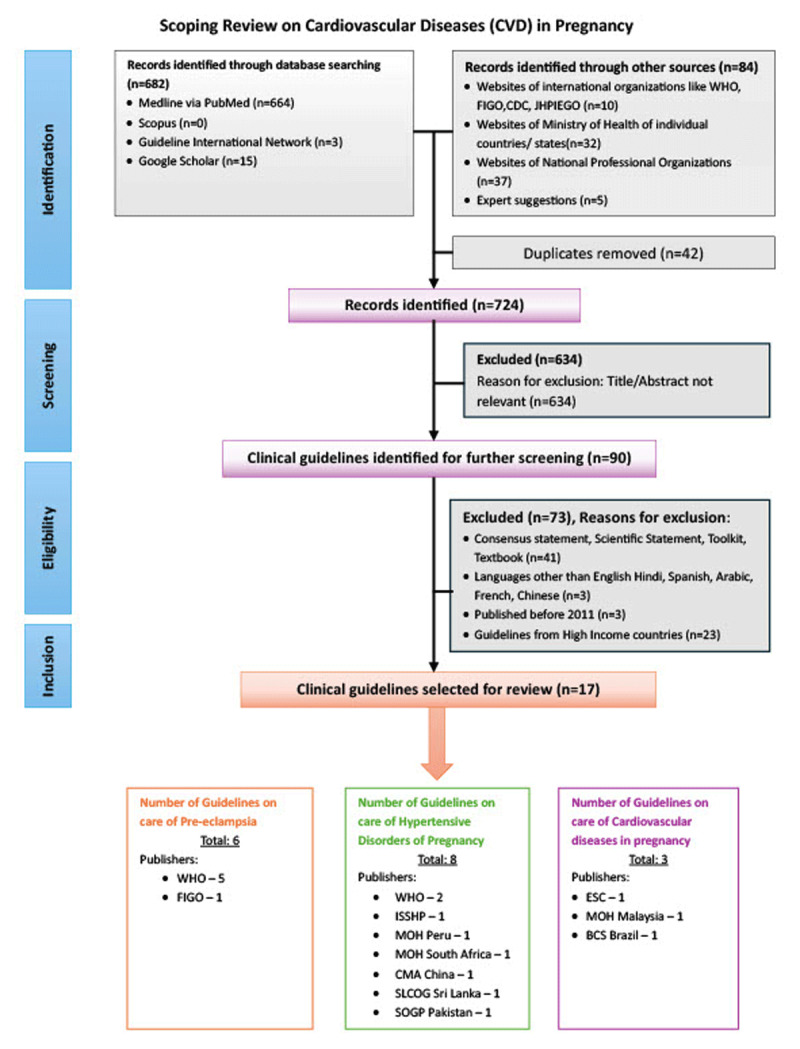
Flowchart presenting the selection of guidelines on CVD in pregnancy modified from PRISMA ScR. WHO- World Health Organization, ESC- European Society of Cardiology, FIGO- The International Federation of Gynaecology and Obstetrics, ISSHP- International Society for the Study of Hypertension in Pregnancy, MOH- Ministry of Health, BCS- Brazilian Cardiology Society, CMA- Chinese Medical Association, SLCOG- Sri Lanka College of Obstetricians and Gynaecologists, SOGP- Society of Obstetricians and Gynaecologists Pakistan.

The scoping review (16) followed the methodology of the Joanna Briggs Institute (JBI) guidelines for scoping reviews (17), which are designed to map the existing evidence on a topic irrespective of study quality or risk of bias. In line with JBI methodology, critical appraisal of individual sources of evidence, including risk of bias assessment, was not conducted, as the aim of a scoping review is to provide an overview of the breadth and nature of available guidance rather than to evaluate the effectiveness or quality of specific interventions. It follows the Preferred Reporting Items for Systematic Reviews and Meta-Analyses extension for Scoping Reviews (PRISMA-ScR) framework (see Figure 1).

### Search strategy

We conducted a systematic search of the electronic databases of PubMed, Scopus, Google Scholar, the GIN (Guidelines International Network) international guideline library (EBSCO), along with individual websites of national and international professional organizations and associations, and Ministries of Health of Low- and Middle-Income countries.

The search strategy aimed to locate both government and non-government guidelines around CVD in pregnancy. An initial limited search of PubMed and Google Scholar was undertaken to identify any guidelines published by professional or other groups. The text words contained in the titles and abstracts of relevant articles and the index terms used to describe the articles were used to develop a full search strategy for databases. The search strategy, including all identified keywords and index terms, was adapted for each included database and/or information source. The search strategy adopted for the database search is given in Annexure 1.

The reference lists of all included sources of evidence were screened for additional documents. The focus of the scoping review was on LMIC; however, only a limited number of sources from LMIC were identified from the database search of published articles. Therefore, a separate search for relevant guidelines was conducted by accessing Ministry of Health websites, country-specific professional organizations, and NGOs for each low- and middle-income country as listed on the World Bank website at the time of the search.

We included clinical guidelines published in English, Spanish, Arabic, and Chinese. Guidelines in Spanish and Chinese were translated into English language with the help of translators and Google Translate. The review was limited to documents published between 2011 and 2023.

### Eligibility criteria

#### Inclusion and exclusion criteria

**Table:** 


Category	Inclusion Criteria	Exclusion Criteria

Population (organizations)	World Health Organization (WHO) International professional organizations* with global reach (e.g., FIGO, ISSHP, and ESC) National professional organizations** in LMICs (e.g., national obstetrics or cardiology associations) Ministries of Health in LMICs	National professional organizations in HICs

Concept	Clinical guidelines addressing cardiovascular diseases (CVDs) before, during, or in the postpartum period—CVDs include cardiac conditions (congenital/acquired) such as valvular diseases, arrhythmias, cardiomyopathies, and ischemic heart disease Hypertensive disorders, including HDP (gestational hypertension, preeclampsia, HELLP syndrome, and eclampsia) and essential hypertension	Guidelines not focused on CVDs in relation to pregnancy

Context	Guidelines developed for or applicable to low- and middle-income countries (LMICs), as per World Bank classification (18) at the time of search	Guidelines solely for high-income countries (HICs)

Types of sources	Clinical practice guidelines summarizing evidence or offering expert recommendations for clinical care	Primary research studies (e.g., clinical trials, cohort or case-control studies)—Conference abstracts


*The international professional organizations included associations, societies, or organizations of professionals with international presence, such as the International Federation of Gynecology and Obstetrics (FIGO), the International Society for the Study of Hypertension (ISSHP), and the European Cardiology Society (ESC), to reach both high-income country (HIC) and low-middle income country (LMIC) populations.

**The national professional organizations included associations, societies, or organizations of obstetrics or cardiology professionals operating within the LMICs.

### Source of evidence selection

Following the search, all identified citations and guidelines were collated and uploaded into a reference management software (Zotero), and duplicates were removed. Titles and abstracts fitting the study description were extracted by one team member. The guidelines that are not meeting the inclusion criteria were excluded. After the preliminary screening of titles/abstracts, the full text of the shortlisted guidelines was reviewed by a team member for relevance. If more than one guideline was available from the same organization, the latest updated version was included. Reasons for exclusion are reported in the flowchart (Figure 1). If the guideline included in the review referred to an older version while discussing recommendations, we have also included the specific content of the recommendation from that guideline. In case of any doubt, the first author referred the concerned guideline to the co-authors and decided the inclusion based on their inputs.

### Data extraction and synthesis of results

Each guideline was first read and summarized. Data were extracted using a data extraction form in MS Excel that was designed for this study. Data items extracted include the title of the guideline, year and type of publication, publishing entity, country of origin, target clinical conditions, and timing and summary of recommendations. The data was extracted by one researcher (UV) and checked for accuracy by another researcher (PR). Clinical guidelines were grouped in terms of geographic location and publisher (WHO, international professional organizations, Ministry of Health, and national professional organizations).

Data are presented as a narrative synthesis, with comparative tables constructed for key areas of practice where differences in recommendations were found.

## Results

### Search outcomes

The search identified 766 documents, including guidelines, research studies, reviews, scientific statements, toolkits, and textbooks. Out of these, 682 were obtained through online database searching, and 84 were identified through searches of individual websites and expert suggestions. After the final screening, 90 documents were considered for inclusion, with 17 meeting the inclusion criteria. Figure 1 summarizes the selection process.

### Characteristics of the guidelines

#### Number and publishers of the guidelines addressing CVD in pregnancy and HDP

Of the 17 clinical guidelines (Figure 1) seven (19,20,21,22,23,24,25) were published by the WHO, three by International Professional Organizations (IPO) (4,26,27), three from Ministries of Health (MOH) (28,29,30) and four from National Professional Organizations (NPO) (31,32,33,34). The full list of guidelines included in the review can be found in Supplementary Table 1.

All the guidelines published by the WHO and IPOs were from organizations headquartered in high-income countries. Four national professional organizations (Brazil, China, Pakistan, and Sri Lanka) and three MOH (Malaysia, Peru, and South Africa) from LMICs, respectively, have published clinical guidelines.

### Scope of the guidelines

Six guidelines addressed preeclampsia exclusively, whilst eight addressed HDP more broadly. Only three guidelines were identified addressing other CVD (including HDP) (Table 1).

**Table 1:** Scope of guidelines on HDP and CVD in pregnancy.


PUBLISHER	TYPE OF PUBLISHER	CLINICAL CONDITION FOCUSED	STAGE OF PREGNANCY FOCUSED	AIM OF THE GUIDELINE RECOMMENDATIONS (PREVENTION/SCREENING/MANAGEMENT)

WHO (19,20,21,22,23,24,25)	WHO	Preeclampsia, Hypertension	Preconception; Antenatal	Prevention; Management

ESC (4)	IPO	CVD	Preconception; Antenatal; Intrapartum; and Postpartum	Prevention; Screening; Management

FIGO (26)	IPO	Preeclampsia	Antenatal	Prevention; Screening

ISSHP (27)	IPO	HDP	Antenatal; Intrapartum; Postpartum and long-term follow-up	Prevention; Screening; Management

MOH, Malaysia (28)	MOH	CVD	Preconception; Antenatal; Intrapartum; Postpartum and long-term follow-up	Prevention; Screening; Management

MOH, Peru (30)	MOH	HDP	Antenatal; Intrapartum	Prevention; Management

MOH, South Africa (29)	MOH	HDP	Antenatal; Intrapartum; Postpartum and long-term follow-up	Prevention; Screening; Management

BCS, Brazil (32)	NPO	CVD	Preconception; Antenatal; Intrapartum and Postpartum	Prevention; Screening; Management

CMA, China (31)	NPO	HDP	Preconception; Antenatal; Intrapartum; Postpartum and long-term follow-up	Prevention; Screening; Management

SLCOG, Sri Lanka (34)	NPO	HDP	Preconception; Antenatal; Intrapartum and Postpartum	Prevention; Screening; Management

SOGP, Pakistan (33)	NPO	HDP	Preconception; Antenatal; Intrapartum; Postpartum and long-term follow-up	Prevention; Screening; Management


CVD, cardiovascular diseases; HDP, hypertensive diseases of pregnancy. WHO, World Health Organization; ESC, European Society of Cardiology; FIGO, The International Federation of Gynaecology and Obstetrics; ISSHP, International Society for the Study of Hypertension in Pregnancy; MOH, Ministry of Health; BCS, Brazilian Cardiology Society; CMA, Chinese Medical Association; SLCOG, Sri Lanka College of Obstetricians and Gynaecologists; SOGP, Society of Obstetricians and Gynaecologists Pakistan.

The preeclampsia guidelines differed in their focus, with the most significant gaps around prevention and screening. This was also observed in the HDP guidelines, with the WHO guidelines only addressing management of HDP, with no recommendations on prevention or screening.

Three guidelines recommended comprehensive care of cardiac diseases in pregnancy. These included screening, prevention, and management of arrhythmia, aortic diseases, cardiomyopathy, congenital heart diseases (CHD), coronary artery disease (CAD), heart failure, pulmonary hypertension, and valvular diseases.

Comprehensive recommendations across the continuum of preconception to postnatal care, with longer-term follow-up of women after the postnatal period, were specified in five guidelines. The HDP guidelines by CMA China, SLCOG Sri Lanka, and SOGP Pakistan contained information about preconception care. The guidelines from ISSHP, South Africa, China, and Pakistan contained information about longer-term care for HDP.

### Recommendations for women with HDP

#### Pharmacological prevention of HDP

Aspirin and calcium are recommended by the guidelines to prevent HDP. However, we observed several differences in the recommendations around its dose (Table 2), duration of aspirin administration during pregnancy (Table 3), and clinical indications for calcium.

**Table 2:** Recommendations for dosage of aspirin and calcium by the guidelines to prevent preeclampsia during pregnancy.


PUBLISHER	ASPIRIN DOSE (mg)	CALCIUM DOSE (g)

WHO	75	1.2–2

ESC	100–150	1.5–2

FIGO	150	1.5–2

ISSHP	150	1.2–2.5

MOH, Malaysia	75	Calcium not recommended

MOH, Peru	100–150	2

MOH, South Africa	75–162	0.5

BCS, Brazil	75–150	1.5–2

CMA, China	50–150	1

SLCOG, Sri Lanka	150	1

SOGP, Pakistan	75–150	Calcium not recommended


WHO, World Health Organization; ESC, European Society of Cardiology; FIGO, The International Federation of Gynaecology and Obstetrics; ISSHP, International Society for the Study of Hypertension in Pregnancy; MOH, Ministry of Health; BCS, Brazilian Cardiology Society; CMA, Chinese Medical Association; SLCOG, Sri Lanka College of Obstetricians and Gynaecologists; SOGP, Society of Obstetricians and Gynaecologists Pakistan.

**Table 3:** Recommended timing and duration of Aspirin administration during pregnancy by the guidelines.


PUBLISHER	COMMENCEMENT TIME OF ASPIRIN ADMINISTRATION (IN WEEKS OF GESTATION)	TIME OF CESSATION OF ASPIRIN ADMINISTRATION (IN WEEKS OF GESTATION)

WHO	20 weeks, or as soon as antenatal care started	Not mentioned in the guideline

ESC	12	36–37

FIGO	11–14	36 or until preeclampsia is diagnosed

ISSHP	Before 16, definitely before 20 weeks	Delivery

MOH, Malaysia	12	Delivery

MOH, Peru	12–16	Not mentioned in the guideline

MOH, South Africa	12–14 (up to 20)	Not mentioned in the guideline

BCS, Brazil	12–16	5 days before delivery

CMA, China	12–16	26–28

SLCOG, Sri Lanka	Before 16	36 weeks

SOGP, Pakistan	13	Delivery


WHO, World Health Organization; ESC, European Society of Cardiology; FIGO, The International Federation of Gynaecology and Obstetrics; ISSHP, International Society for the Study of Hypertension in Pregnancy; MOH, Ministry of Health; BCS, Brazilian Cardiology Society; CMA, Chinese Medical Association; SLCOG, Sri Lanka College of Obstetricians and Gynaecologists; SOGP, Society of Obstetricians and Gynaecologists Pakistan.

The dose of aspirin recommended for preeclampsia prevention varied from 50 to 160 mg. Two MOH guidelines from Peru and South Africa (30,35) and three NPO guidelines—CMA China, BCS Brazil, and SOGP Pakistan (31,32,33)—offered a range of aspirin dosages, presumably reflecting locally available formulations. In addition to variations in the dose, there was significant heterogeneity regarding the weeks of pregnancy at which aspirin was recommended to commence and end (Table 3).

The recommended dose of calcium also varied from 500 mg by MOH South Africa to 2500 mg by ISHHP, with the ESC and FIGO citing an acceptable range of 1.5–2 g, acknowledging the availability of different formulations depending on the country and may be to a certain extent reflect the uncertainty regarding dosage and prophylactic efficacy (Table 2). Other differences in recommendations were also noted, with the MOH, Malaysia and SLCOG, Sri Lanka, not recommending calcium at all, whilst the MOH, South Africa, recommending universal supplementation. Five of the guidelines (4,20,26,27,32) recommend using dietary thresholds to determine which women should receive calcium supplementation, whereas two guidelines (30,33) recommend calcium only for women at high risk for HDP.

### Recommendations on the pharmacological management of HDP

All 12 guidelines recommending antihypertensive medications provided separate recommendations for non-severe hypertension and severe hypertension.

Eleven guidelines recommend commencing antihypertensive treatment for non-severe hypertension when blood pressure exceeds or is equal to 140/90 mmHg. The WHO guideline, on the other hand, recommends initiating treatment at or above 100 mmHg diastolic.

All guidelines recommended either α-methyldopa, labetalol, or nifedipine for the first-line management of HDP. However, the recommended order varied between guidelines. Of note, only one guideline (i.e., ISHHP) specified that α-methyldopa or nifedipine can be used in low-resource settings for managing HDP.

Guidelines published by IPOs (ESC and ISSHP), MOH (Malaysia, Peru, and South Africa) and NPOs (BCS Brazil, CMA China, SLCOG Sri Lanka, and SOGP Pakistan) recommend using a blood pressure threshold of 160/110 mmHg to define severe hypertension. Furthermore, these guidelines specified that this threshold is to be considered as an acute emergency to start rapid blood pressure control. In contrast, the WHO did not define a threshold for ‘severe hypertension’ but recommends the use of antihypertensive medications for managing severe hypertension during pregnancy.

Box 1 summarizes the medications and treatment recommended for managing severe hypertension during pregnancy.

**Box 1 Summary of recommendations for managing severe hypertension during pregnancy**

- *Nifedipine* is recommended by WHO, IPO (ESC and ISSHP), MOH (Malaysia, Peru, and South Africa), NPO (BCS Brazil, CMA China, SLCOG Sri Lanka, and SOGP Pakistan) as medication for rapid control of blood pressure in emergencies. All ten guidelines recommend a dosage of 10 mg oral nifedipine. The BCS Brazil also recommends that sublingual administration of quick-acting nifedipine should be avoided due to the risk of hypotension.
- *Intravenous administration of labetalol* is also recommended by the above-mentioned guidelines for rapid control of blood pressure in pregnant women. The ISSHP guideline recommends the use of oral route along with the intravenous (IV) route for the administration of labetalol. The ESC and BCS Brazil recommend intravenous administration of labetalol as the first line of drug for controlling the acute onset of hypertension during pregnancy and the postpartum period. The MOH (South Africa), however, recommends labetalol as a second-line antihypertensive drug. All guidelines recommend a dosage of 20–150 mg/hr.
- *Intravenous administration of hydralazine* is recommended by the WHO, ISSHP, MOH (Malaysia), and BCS Brazil as the second line of treatment for rapid control of severe hypertension and preeclampsia. The ESC guideline prohibits the use of hydralazine as the drug of choice for controlling hypertension due to its adverse effects on perinatal health.
- *Nitroglycerin* (glyceryl trinitrate) is recommended by ESC, BCS Brazil, and CMA China for controlling preeclampsia-associated acute pulmonary edema.
- *Sodium nitroprusside* is recommended by ESC, BCS Brazil, and CMA China only in extreme situations to avoid the risk of fetal cyanide poisoning.
- The other drugs recommended by ESC, ISSHP, MOH Malaysia, and CMA China guidelines include uradipil, prazosin, nimodipin, phentolamine and nicardipine.

Six guidelines highlighted antihypertensives contraindicated during pregnancy due to potential fetal risk (WHO, ESC, CMA China, BCS Brazil, SLCOG Sri Lanka, and SGP Pakistan). These include angiotensin-converting enzyme inhibitors (ACE-I) and angiotensin receptor blockers (ARB) due to the risk associated with renal damage in the fetus. All six guidelines suggest switching to safer options as soon as a pregnancy is confirmed.

Conflicting recommendations were observed between guidelines, notably for using thiazide diuretics and sodium nitroprusside during pregnancy. WHO prohibits the use of sodium nitroprusside in pregnancy, whereas ESC, CMA China, and BCS Brazil gave cautious recommendations for extreme situations such as refractory hypertension or severe hypertension with risk of death. Similarly, SOGP Pakistan and MOH Malaysia prohibit the use of thiazide diuretics in pregnancy; however, BCS Brazil recommends it as a second-line antihypertensive medication.

### Recommendations for non-pharmacological management of HDP

Dietary and lifestyle changes in women with HDP were recommended by seven guidelines (Table 4).

**Table 4:** Non-pharmacological interventions recommended for the management of HDP.


TYPE OF PUBLISHER	PUBLISHER	NON-PHARMACOLOGICAL INTERVENTIONS					

		SALT RESTRICTION	VITAMIN SUPPLEMENTATION	PHYSICAL ACTIVITY	ALCOHOL RESTRICTION	TOBACCO RESTRICTION	RISK COUNSELING

	WHO	–	–	–	–	–	–

International Professional Organization	ESC	–	×	✓	✓	✓	✓

	FIGO	–	–	–	–	–	–

	ISSHP	–	×	✓	–	–	–

Ministry of Health	MOH, Malaysia	–	✓	✓	✓	✓	✓

	MOH, Peru	–	–	–	–	–	–

	MOH, South Africa	–	–	–	–	–	–

National Professional Organization	BCS, Brazil	×	×	✓	✓	✓	✓

	CMA, China			✓	✓	✓	✓

	SLCOG, Sri Lanka	×	–	✓	–	–	✓

	SOGP, Pakistan	✓	✓	✓	✓	✓	✓


Legend: ✓, recommended; ×, not recommended; –, no recommendation available in the guideline. WHO, World Health Organization; ESC, European Society of Cardiology; FIGO, The International Federation of Gynaecology and Obstetrics; ISSHP, International Society for the Study of Hypertension in Pregnancy; MOH, Ministry of Health; BCS, Brazilian Cardiology Society; CMA, Chinese Medical Association; SLCOG, Sri Lanka College of Obstetricians and Gynaecologists; SOGP, Society of Obstetricians and Gynaecologists Pakistan.

All seven guidelines recommend physical activity in the form of mild and moderate intensity exercise with counseling of pregnant women along with their family members about the associated risks of CVD/HDP in pregnancy. Five of these guidelines recommend restricting the consumption of alcohol and tobacco.

SOGP Pakistan recommends restricting salt intake by pregnant women with HDP, however, SLCOG Sri Lanka and BCS Brazil recommend against this, citing a lack of evidence in the pregnant population.

The guidelines also differed on vitamin supplementation recommendations. MOH Malaysia and SGOP Pakistan recommend vitamin supplementation for pregnant women with HDP, while the ESC, ISSHP, and BCS Brazil guidelines prohibit Vitamin C and E supplementation, citing its potential harmful effects on pregnant women with HDP.

### Recommendations for women with cardiac disease in pregnancy

#### Pre-conception care: Preparing for pregnancy in women with known heart disease

Of the three guidelines on cardiac disease in pregnancy identified—ESC (4), MOH Malaysia (28), and BCS Brazil (15)—all recommend pre-conception counseling for women who are diagnosed with heart disease and planning for pregnancy.

#### Antenatal care

All three guidelines recommend using the WHO classification of maternal cardiovascular risk and the New York Heart Association (NYHA) functional classification to assess maternal risk. The ESC further recommends using predictors identified by studies such as CARPREG (Cardiac disease in Pregnancy). All three guidelines recommend baseline investigations of an ECG and echocardiography in patients with cardiac disease, with the ESC additionally recommending exercise testing.

#### Antibiotic use in pregnancy with cardiac disease

Antibiotic use in pregnancy is found to vary across three guidelines. While all three guidelines agreed upon the therapeutic use of antibiotics in conditions such as endocarditis and rheumatic fever (for secondary prophylaxis), prophylactic use of antibiotics was not universally recommended. The ESC specifies that antibiotic prophylaxis should not be used in pregnancy, while the MOH Malaysia and BCS (Brazil) guidelines recommend prophylactic antibiotics during delivery, specifically ampicillin and gentamicin.

#### Cardiac interventions during pregnancy

The guidelines recommended percutaneous therapy and surgical intervention after 4 months of gestation. Cardiac surgery is recommended only if medical therapy or interventional procedures fails, and the mother’s life is threatened.

#### Anticoagulation during pregnancy with cardiac disease

Whilst the guidelines contained some differences in the cardiac conditions for anticoagulation (prophylactic or therapeutic), in women with mechanical heart valves, the highest risk group for thrombus during pregnancy, guidance was broadly similar. This was centered around discussing the risks and benefits of vitamin K agonists, unfractionated or low molecular weight heparin, with the woman and her family. In women conceiving on low-dose warfarin (<5 mg), the risk of congenital malformations was deemed low, and continuation may be considered. Women on higher doses of warfarin should switch to low molecular weight heparin (if Xa monitoring is available) or IV unfractionated heparin for the first trimester only. During the second and third trimesters, all guidelines recommend warfarin until 36 weeks. After 36 weeks, and around the delivery, the guidelines recommended either LMWH or IV unfractionated heparin, with minor differences in the timing of cessation before birth and recommencement after birth (Supplementary Table 2)

### Intrapartum care for women with CVD

#### Time of delivery

Consultation with the multidisciplinary team led by the Obstetrician and Cardiologist to assess the woman’s cardiac condition and fetal wellbeing and decide the time of delivery is recommended. The guidelines also recommend considering induction of delivery at 40 weeks of gestation for all women with heart disease. Additionally, for women taking vitamin K antagonists, MOH Brazil recommends delivery from 37 weeks of gestation.

#### Route of delivery

Vaginal delivery is the recommended route of delivery for pregnant women with uncomplicated or non-severe cardiac conditions. The indications for cesarean section are cardiac conditions of the severity of NYHA/WHO class 3 and 4, severe obstructive cardiac lesions, pulmonary hypertension, including Eisenmenger’s syndrome, severe aortic pathologies, acute heart failure, peripartum cardiomyopathy, and patients on oral anticoagulants. All three guidelines recommend a lateral decubitus position for delivery. Assisted delivery by shortening the 2nd stage, either by vacuum extraction or forceps, is also recommended.

#### Maternal monitoring during delivery

The guidelines recommend continuous monitoring of maternal BP and heart rate during the delivery of women with cardiac disease. The guidelines further recommend the use of pulse oximetry and ECG to detect early signs of decompensation.

#### Postpartum care and long-term follow-up

All three guidelines on CVD in pregnancy recommend hemodynamic monitoring of women after delivery. Oxytocin and misoprostol are recommended as the drugs of choice to prevent PPH, while thromboprophylaxis using UFH/LMWH and meticulous leg care along with early ambulation are suggested for preventing venous thrombosis.

#### Multispecialty involvement in the care of patients with heart disease in pregnancy

The need to have a multidisciplinary approach for pregnancy care with cardiovascular disease and specialist care involving cardiologists based on the risk of CVD is recommended by three guidelines.

The ESC guideline further recommends a ‘pregnancy heart team’ for women with moderate/high risk (mWHO II–III, III, and IV) for pre-pregnancy counselling and management during pregnancy and postpartum.

## Discussion

This scoping review, to our knowledge, is the first to identify and compare clinical guidelines related to CVD in pregnancy that are applicable to LMICs. The findings highlight several important gaps in the geographic and population coverage of locally produced and contextually relevant guidelines for CVD in pregnancy for LMICs. Despite an extensive search, only 17 guidelines from the past 12 years were identified. Of these guidelines, only 7 were from LMICs, and 10 guidelines were published by international organizations. This highlights the lack of focus on cardiovascular disease (CVD) in pregnancy in LMICs.

This review also reveals a skewed focus, with 14 guidelines concentrating on preeclampsia or hypertensive disorders of pregnancy (HDP). Only three guidelines were identified addressing the complex needs of women who enter pregnancy with cardiac disease, one from the European Society of Cardiology, and two from national organizations in middle-income countries (Brazil and Malaysia).

The findings highlight significant heterogeneity in the content of the guidelines, such as those in the prevention and management of preeclampsia/HDP, and differences in the prevention of endocarditis in women with cardiac disease. Some of these differences may be due to the guideline’s publication date and subsequent updating or publication of new evidence. For example, the optimal recommended dose of aspirin was revised in 2017 from 75 mg daily to 150 mg daily based on new analysis of the dose–response effect of aspirin as a prophylactic agent. The differences in recommendations among guidelines from LMICs could be influenced by the local burden of disease, healthcare infrastructure, and the availability of evidence specific to these regions. Therefore, while global guidelines may provide a framework, there is a need for context-specific adaptations in LMICs to ensure effective prevention and management strategies for pregnant women with cardiovascular disease.

One such example of limited contextual relevance is the treatment of Rheumatic Heart Disease (RHD), a leading cause of cardiac morbidity in pregnancy in LMICs. While MOH Malaysia (28) does not mention RHD at all, even in the section of valvular lesions, the ESC guideline (4) discusses RHD under broader valvular heart disease categories, focusing mainly on mitral stenosis but without acknowledgement of the epidemiological and health systems implications unique to LMICs. In contrast, BCS Brazil (32) offers a more comprehensive and context-sensitive approach, recognizing RHD as a key maternal cardiovascular issue. It includes detailed guidance on risk stratification, timing of intervention (e.g., balloon mitral valvotomy), anticoagulation, and multidisciplinary management. BCS Brazil also contains a screening and treatment algorithm for women with Chagas disease, prevalent in some regions in South America, highlighting the need for context-specific guidance.

Our findings resonate strongly with the work of Sliwa and colleagues in South Africa, who have shown that RHD, along with peripartum cardiomyopathy and uncorrected congenital heart lesions, constitutes a significant proportion of maternal cardiovascular deaths in LMICs (36). Sliwa advocates for a model of integrated, context-specific care built around early detection, risk stratification, and multidisciplinary collaboration, including obstetricians, cardiologists, and community health workers (36,37). The recent scientific statement on peripartum heart failure by the Heart Failure Association of the European Society of Cardiology (38) emphasizes the critical role of coordinated multidisciplinary care in improving clinical outcomes for pregnant women with cardiovascular disease. These lessons are particularly relevant for LMICs where delayed diagnosis, limited access to echocardiography, and fragmented referral pathways contribute to preventable maternal deaths.

In addition, guidelines such as the 2022 Australian RHD guideline (39) provide a public health-oriented framework that could inform LMIC adaptations. It emphasizes a life course approach to RHD addressing primordial prevention (e.g., skin and throat infections), early diagnosis in adolescents, secondary prophylaxis, reproductive planning, and tailored antenatal care. Such a continuum of care model is currently absent in most global and national CVD in pregnancy guidelines.

The variation in recommended pharmacologic management of HDP and severe HDP suggests the limited availability of well-conducted trials to inform clinical decision-making in this area. Most antihypertensives used in pregnancy are used ‘off label’, meaning that safety and efficacy trials in pregnancy have not been done, or the manufacturers have not applied for a specific license in pregnancy. The licensing of drugs for use in pregnancy is problematic globally, with little motivation for pharmaceutical companies to market drugs for pregnancy following the historical tragedy of thalidomide. The need to include pregnant women in clinical trials and develop drugs specifically for pregnancy has been highlighted globally (40,41). The unique health profiles of LMIC populations, including higher rates of malnutrition, infections, and comorbidities, underscore the importance of developing pregnancy-specific drugs that address these contexts. Without inclusive clinical trials that involve pregnant women from diverse backgrounds, even the scarcely available global evidence base remains skewed toward high-income settings, making it less applicable to LMICs. Addressing this gap requires global and local efforts to advocate for the inclusion of pregnant women from LMICs in research and increased funding for pregnancy-specific drug development.

Developing contextually appropriate clinical guidelines for resource-constrained settings is challenging, balancing the desire to provide the best evidence-based and highest-quality care against local resource constraints. Having said that the need for guidelines for LMICs cannot be negated as this limitation can hinder timely and effective treatment of conditions such as preeclampsia, leading to potentially life-threatening complications for both the mother and the baby, and can contribute to the global burden of maternal deaths. It was positive to see the ISSHP guideline, an international society, including specific medication recommendations for low-income settings for the management of hypertension in pregnancy. This would be a welcome development for other international societies in cardiology to consider in future guidelines, particularly around the management of cardiac conditions seen more commonly in LMICs, such as rheumatic heart disease, heart failure secondary to severe anemia, and uncorrected or partially corrected congenital heart conditions. Developing these guidelines will require investing on research based in LMICs, and this would be possible through adequate funding along with consultation and partnership with LMIC cardiologists and obstetricians, as well as consumer voices of women and their families recognizing priorities for their health.

## Limitations

First, article and guideline selection were conducted by a single reviewer, without dual independent screening or adjudication by a third reviewer. The data extraction and findings were checked by a second reviewer, and the findings were discussed with clinicians experienced in this area to minimize this bias. Despite searching multiple databases, we may have missed some guidelines due to language limitations (English, Spanish, Chinese, and Arabic) as we lacked the resources for other languages. Our search strategy, relying on databases and public domain documents, might have excluded relevant guidance. The 2011–2023 publication timeframe aimed to reflect contemporary practice, but older guidelines may still be used in many LMICs. Additionally, guideline inclusion does not guarantee implementation. This scoping review’s objective was to map guideline availability and scope, not detailed clinical treatment pathways. Future work, such as systematic reviews or guideline appraisals, may be needed to address clinical decision-making in more detail.

Another limitation of this review is that it lacked stakeholder engagement at the time of protocol development. The review, however, was presented at a webinar cohosted by the Taskforce on Women and NCDs, American Heart Association, and the George Institute for Global Health on 08-02-2024 (https://www.taskforcewomenandncds.org/bridging-the-gaps-in-maternal-heart-health-during-pregnancy/) attended by policymakers and clinicians from around the world, with feedback which has been incorporated into this manuscript. We believe that consultations and focus groups with key LMIC stakeholders, including cardiologists, obstetricians, anesthetists, midwives, and professional society representatives, will continue to help identify context-specific implementation challenges and evidence gaps.

## Conclusion

Despite being a leading cause of maternal mortality globally, clinical guidelines on cardiovascular disease (CVD) in pregnancy remain scarce for healthcare teams in low- and middle-income countries (LMICs). There is an urgent need to understand contextual challenges in caring for women with complex CVD across different geographies and resource levels, specifically for healthcare professionals in LMICs.

## Annexure 1

Search conducted between 04-03-2023 and 04-05-2023.

### 1. PubMed

(‘guideline’[Publication Type] OR ‘Guidelines as Topic’[Majr] OR guideline*[All Fields] OR recommendation*[All Fields]) AND (‘Cardiovascular Diseases’[Mesh] OR ‘Heart Diseases’[Mesh] OR cardiac[All Fields] OR ‘heart disease’[All Fields] OR ‘cardiac disorder’[All Fields] OR ‘Blood Pressure’[Mesh] OR ‘Hypertension’[Mesh] OR ‘Eclampsia’[Mesh] OR ‘Pre-Eclampsia’[Mesh] OR ‘HELLP Syndrome’[Mesh] OR ‘blood pressure’[All Fields] OR hypertension[All Fields] OR eclampsia[All Fields] OR pre-eclampsia[All Fields] OR ‘HELLP syndrome’[All Fields]) AND (‘Pregnancy’[Mesh] OR ‘Gravidity’[Mesh] OR pregnan*[All Fields] OR gestation[All Fields]) AND (‘2011/01/01’[PDAT] : ‘2023/12/31’[PDAT]) AND free full text[sb]

### 2. Scopus

(TITLE-ABS-KEY(guideline) OR TITLE-ABS-KEY(guidelines) OR TITLE-ABS-KEY(recommendation) OR TITLE-ABS-KEY(recommendations)) AND (TITLE-ABS-KEY(cardiac) OR TITLE-ABS-KEY(‘heart disease’) OR TITLE-ABS-KEY(‘heart diseases’) OR TITLE-ABS-KEY(‘cardiac disorder’) OR TITLE-ABS-KEY(‘cardiovascular disease’) OR TITLE-ABS-KEY(hypertension) OR TITLE-ABS-KEY(‘blood pressure’) OR TITLE-ABS-KEY(eclampsia) OR TITLE-ABS-KEY(‘pre-eclampsia’) OR TITLE-ABS-KEY(‘HELLP syndrome’)) AND (TITLE-ABS-KEY(pregnancy) OR TITLE-ABS-KEY(pregnant) OR TITLE-ABS-KEY(gestation) OR TITLE-ABS-KEY(gravidity)) AND (PUBYEAR >2010 AND PUBYEAR <2023)

### 3. Google Scholar, GIN International library, and other individual websites

‘Guidelines’, ‘Recommendations’, ‘Cardiovascular diseases in Pregnancy’, ‘Heart Diseases in Pregnancy’, ‘Hypertensive Disorders of Pregnancy’, ‘Hypertension in Pregnancy’.

## Additional File

The additional file for this article can be found as follows:

10.5334/gh.1453.s1 Supplementary Files. Supplementary Tables 1 and 2.
