# Supplementary material for: A Scoping Review of Clinical Guidelines for the Management of Cardiovascular Diseases (CVD) in Pregnancy in Low- and Middle-Income Countries (LMIC)
Source: Glob Heart. 2025 Aug 21;20(1):69. doi: 10.5334/gh.1453 (PMC12372675; doi:10.5334/gh.1453)
Supplement: Supplementary Files. — Supplementary Tables 1 and 2. [file gh-20-1-1453-s1.pdf]

Supplementary Table.1. List of Guidelines on Cardiovascular Diseases in Pregnancy included in the scoping review

Title of the Guideline	Publisher	Country/ Region	Year of publication	CVD conditions	Target population	
					Pregnant women	Health provider
Clinical Practice Guideline-Heart Disease in Pregnancy (28)	Ministry of Health, Malaysia, Academy of Medicine, Malaysia and National Heart Association of Malaysia	Upper middle income	2016	Valvular Heart Disease, Cardiomyopathy, Arrhythmia, Other structural abnormalities, Coronary Artery Disease, Hypertension, Eclampsia, Pre eclampsia,	Women with cardiac disease planning for pregnancy and pregnant women with underlying cardiac disease	primary care physicians, obstetricians/ gynaecologists, cardiologists/ surgeons,
The hypertensive disorders of pregnancy: ISSHP classification, diagnosis & management recommendations for international practice (27)	International Society for the Study of Hypertension in Pregnancy (ISSHP)	Global/ Regional	2018	Hypertension, Eclampsia, Pre eclampsia,	The target population is not specified in the document, though it include all pregnant women during pregnancy, delivery and after delivery	All clinicians
2018 ESC Guidelines for the management of cardiovascular diseases during pregnancy (4)	The Task Force for the Management of Cardiovascular Diseases during Pregnancy of the European Society of Cardiology (ESC)	Global/ Regional	2018	Valvular Heart Disease, Cardiomyopathy, Arrhythmia, Other structural abnormalities, Coronary Artery Disease, Ischaemic Heart Disease, Hypertension, Eclampsia, Pre eclampsia	All pregnant women already diagnosed or newly diagnosed with CVD.	Not specified as target audience, though elsewhere mentioned addressing all health staff who are involved in specialty care of pregnant women with CVD
The International Federation of Gynecology and Obstetrics (FIGO) initiative on pre-eclampsia: A pragmatic guide for first-trimester screening and prevention (26)	International Federation of Gynecology and Obstetrics	Global/ Regional	2019	Hypertension, Pre eclampsia	Women at the 1st trimester of pregnancy, with or without diagnosed as having Pre eclampsia	Healthcare providers such as community health workers, midwives/nurses, primary care physicians, obstetricians/gynaecologists,
Hypertensive disorders in pregnancy: 2019 National guideline (29)	Ministerial National Committee on Confidential Enquiries into	Upper middle income	2019	Hypertension, Eclampsia, Pre eclampsia	The target population is pregnant women with hypertension at every level of care.	Healthcare professionals

Title of the Guideline	Publisher	Country/ Region	Year of publication	CVD conditions	Target population	
					Pregnant women	Health provider
	Maternal Deaths in South Africa.					
WHO recommendations on drug treatment for non-severe hypertension in pregnancy (21)	The World Health Organization	Global/ Regional	2020	Hypertension, Eclampsia, Pre eclampsia	pregnant women in low-, middle- or high income settings, particularly those who experience non-severe hypertension during pregnancy.	midwives/nurses, primary care physicians, obstetricians/gynaecologists, Healthcare providers, managers of maternal and child health programmes, and relevant staff in ministries of health
WHO recommendations Drug treatment for severe hypertension in pregnancy (22)	The World Health Organization	Global/ Regional	2020	Hypertension, Eclampsia, Pre eclampsia	The population affected by the recommendations includes pregnant women in low-, middle- or high-income settings, particularly those who experience severe hypertension during pregnancy.	midwives/nurses, primary care physicians, obstetricians/gynaecologists, Healthcare providers, Managers, relevant ministry staff
WHO recommendation on Calcium supplementation before pregnancy for the prevention of pre-eclampsia and its complications (19)	The World Health Organization	Global/ Regional	2018	Pre eclampsia	The population affected by this recommendation includes women (particularly those intending to become pregnant and those women at higher risk of gestational hypertensive disorders) in low, middle or high-income settings	midwives/nurses, primary care physicians, obstetricians/gynaecologists, health professionals, program managers, ministry staff
WHO recommendation Calcium supplementation during pregnancy for the prevention of pre-eclampsia and its complications (20)	The World Health Organization	Global/ Regional	2020	Eclampsia, Pre eclampsia	The population affected by this recommendation includes all pregnant women (particularly those at higher risk of gestational hypertensive	midwives/nurses, primary care physicians, obstetricians/gynaecologists, health professionals,

Title of the Guideline	Publisher	Country/ Region	Year of publication	CVD conditions	Target population	
					Pregnant women	Health provider
					disorders) in low-, middle- or high-income settings, and those living in areas where dietary intake of calcium is low	program managers, ministry staff
WHO recommendations Policy of interventionist versus expectant management of severe pre-eclampsia before term (23)	The World Health Organization	Global/ Regional	2018	Pre eclampsia	The population affected by the recommendations includes pregnant women in low-, middle or high-income settings, particularly those who experience severe pre-eclampsia during pregnancy.	midwives/nurses, primary care physicians, obstetricians/gynaecologists, health professionals, managers, ministry staff
WHO recommendations for Prevention and treatment of pre-eclampsia and eclampsia (24)	The World Health Organization	Global/ Regional	2011	Pre eclampsia	The population affected by the recommendations includes pregnant women in low-, middle or high-income settings, particularly those who experience severe pre-eclampsia during pregnancy.	midwives/nurses, primary care physicians, obstetricians/gynaecologists, health professionals, managers, ministry staff
WHO recommendations on antiplatelet agents for the prevention of pre-eclampsia (25)	The World Health Organization	Global/ Regional	2021	Pre eclampsia	The population affected by the recommendations includes pregnant women in low-, middle or high-income settings, particularly those who experience severe pre-eclampsia during pregnancy.	midwives/nurses, primary care physicians, obstetricians/gynaecologists, health professionals, managers, ministry staff
Hypertensive disorders of pregnancy (34)	Sri Lanka College of Obstetricians and Gynaecologists (SLCOG)	Lower middle income	2022	Hypertension, Eclampsia, Pre eclampsia	Not stated specifically, though women before and during pregnancy & during and after delivery	Not specified

Title of the Guideline	Publisher	Country/ Region	Year of publication	CVD conditions	Target population	
					Pregnant women	Health provider
					are targeted at each stage	
Society of Obstetricians and Gynaecologists Pakistan (SOGP) Hypertensive Disorders in Pregnancy Guidelines- 2022 (33)	Society of Obstetricians and Gynaecologists Pakistan (SOGP)	Lower middle income	2022	Hypertension, Eclampsia, Pre eclampsia	Not stated though women intending to become pregnant, pregnant women during antenatal period, during delivery and after delivery are considered	All health professionals
Diagnosis and treatment of hypertension and pre-eclampsia in pregnancy: a clinical practice guideline in China（2020) (31)	Chinese Society of Obstetrics and Gynecology, Chinese Medical Association	Upper middle income	2020	Chronic hypertension	Women with underlying cardiovascular diseases or medical conditions; women with previous history of preeclampsia, multiple pregnancy	All clinicians
Brazilian Cardiology Society Statement for Management of Pregnancy and Family Planning in Women with Heart Disease – 2020 (32)	Brazilian Cardiology Society	Upper middle income	2020	Congenital Heart Disease, Valvular Heart Disease, cardiomyopathy, Ischemic Heart Disease, Pulmonary HTN, Aortic HTN, Arrhythmia, Pulmonary HTN, Aortic Disease, Hypertensive Diseases in Pregnancy, Heart Failure	Pregnant women with heart disease	Not specified
Guía de práctica clínica para la prevención y el manejo de la enfermedad hipertensiva del embarazo- Clinical practice guideline for the prevention and management of hypertensive disorders of pregnancy (30)	Social Health Security (EsSalud) of Peru	Upper middle income	2022	Hypertension, Eclampsia, Pre eclampsia	The target population of the guide are pregnant women at risk of developing or diagnosed with EHE, preeclampsia and severe preeclampsia.	clinicians at all levels of care providing care for pregnant women

Supplementary Table.2.

Recommendations for Anticoagulation from the guidelines on CVD in pregnancy

Guideline	Mechanical heart valves	Delivery for those with mechanical heart valves	Other indications
MoH Malaysia	• Discussion with patient and family.<br>• Warfarin advocated throughout pregnancy up to 36 weeks<br>• Alternate regime warfarin in the 2nd and 3rd trimester with heparin based therapy (unfractionated heparin or LMWH) in the 1st TM.<br>• From 36 weeks: LMWH or UFH	• Women on LMWH switched to IV UFH at least 36 hours before induction of planned caesarean section. Discontinued 4-6 hours before delivery and restarted 4-6 hours (vaginal) or 6-12 hours after caesarean.<br>• Oral anticoagulation resumed after 24 hours if no bleeding concerns	• Choice dependent on risk of thrombosis, patient preference, and consensus of multidisciplinary team.<br>• Regime A – warfarin throughout pregnancy until 36 weeks<br>• Regime B – UFH or LMWH in 1st trimester and warfarin in the 2nd and 3rd trimester
ESC	• Pre pregnancy counselling<br>• Continuation of VKA (vitamin K Agonist) throughout pregnancy if dose is low<br>• Alternately switch to LMWH from weeks 6-12 or UFH with regular monitoring<br>From 36 weeks:<br>• LMWH or UFH<br>• 36 hours before delivery:- IV UFH	• For women with planned CS, LMWH omitted for 24 hours prior to surgery<br>• High-risk women UFH started 6 hours after delivery<br>• Vaginal delivery moderate to high-risk patients converted to an infusion of UFH, with the infusion stopped 4-6 hours prior to regional anaesthesia or anticipated birth	• Anticoagulation prophylaxis or therapeutic recommendations (depending on risk of thrombus) for native valvular heart disease, Fontan circulation, pulmonary arterial hypertension, mitral stenosis, atrial fibrillation, heart failure during pregnancy (PPCM and DCM), persistent or paroxysmal arrythmias in hypertrophic cardiomyopathy.

		• Low risk women, LMWH omitted for 24 hours prior to anticipated delivery<br>• Urgent delivery in women on UFH, protamine sulphate can be given. Longer courses needed for LMWH.<br>• In women on OAC requiring urgent reversal, four-factor prothrombin complex concentrate recommended prior to CS. Fetus may need fresh frozen plasma as well as vitamin K	
BCS Brazil	• 1st trimester substituting VKA with LMWH (if weekly Xa monitoring available), IV UFH from 6th-9th week<br>• Replace LMWH or IV UFH with VKA<br>• Week 36: hospitalization. Change to IV UFH or LMWH<br>• 24 hours before delivery- Maintain UFH IV	• Withdraw UFH 4-6 hours before delivery and restart 4-6 hours afterwards if no bleeding. Reintroduce VKA 48 hours after delivery with IV UFH	Pulmonary hypertension and Eisenmenger syndrome (prophylactic dose), noncompaction cardiomyopathy, dilated cardiomyopathy, intracavitary thrombus or prior embolic event, PPCM, systemic vasculitis
